# The clinical effect of arrhythmia monitoring after myocardial infarction (BIO-GUARD|MI):study protocol for a randomized controlled trial

**DOI:** 10.1186/s13063-019-3644-5

**Published:** 2019-09-11

**Authors:** Christian Jons, Peter Sogaard, Steffen Behrens, Jürgen Schrader, Sascha Mrosk, Poul Erik Bloch Thomsen

**Affiliations:** 1 Department of Cardiology, Rigshospitalet, Blegdamsvej 9, DK-2100 Copenhagen, Denmark; 2 Department of Cardiology, Aalborg University Hospital, Søndre Skovvej 15, DK-9000 Aalborg, Denmark; 3 Vivantes Humboldt Klinikum, Abteilung für Kardiologie und konservative Intensivmedizin, Am Nordgraben 2, 13509 Berlin, Germany; 4 Biotronik SE & Co. KG, Woermannkehre 1, 12359 Berlin, Germany

**Keywords:** Implantable cardiac monitor, Cardiac arrhythmia, Myocardial infarction, Major adverse cardiac event, CHADS-VASC score

## Abstract

**Background:**

The increasing use of implantable cardiac monitors (ICMs) allows early documentation of asymptomatic cardiac arrhythmias that would previously have gone unnoticed. The addition of remote monitoring to cardiac devices means that physicians receive an early warning in cases of new-onset arrhythmias. While remote monitoring has been suggested to increase survival in heart failure patients with implantable defibrillators, trials using ICMs for continuous electrocardiographic monitoring of cardiac arrhythmias in the postmyocardial infarction setting have shown that patients who experienced cardiac arrhythmias such as atrial fibrillation, bradycardia, and ventricular tachyarrhythmia have an increased risk of major adverse cardiac events.

**Methods:**

The Biomonitoring in patients with preserved left ventricular function after diagnosed myocardial infarction (BIO-GUARD-MI) study is designed to investigate and clarify whether the incidence of major adverse cardiac events can be decreased by early detection and treatment of cardiac arrhythmias using an ICM in patients after myocardial infarction. In addition, the study will allow us to describe the interplay between baseline characteristics, arrhythmias, and clinical events to improve the treatment of this high-risk patient population. The study will enroll and randomize a cohort of high-risk postmyocardial infarction patients with CHA_2_DS_2_-VASc score ≥ 4 and left ventricular ejection fraction > 35% to an ICM or conventional treatment. Physicians are provided with suggestions on how to respond to ICM-documented arrhythmias. An estimated 1400 patients will be enrolled and followed until 372 primary endpoints have occurred. In this paper, we describe the literature and rationale behind the design and interventions towards new-onset arrhythmias, as well as future perspectives and limitations for the use of ICMs.

**Discussion:**

Remote monitoring may improve clinical outcome if it uncovers conditions with low symptom burden which cause or indicate an increased risk. A simple and easily implementable response to the information is important. Cardiac arrhythmias frequently start as asymptomatic, shorter lasting, and nightly events. The BIO-GUARD-MI trial represents the first attempt to simplify the response to the rather complex nature of heart arrhythmias.

**Trial registration:**

Clinical Trials, NCT02341534. Registered on 19 January 2015.

**Electronic supplementary material:**

The online version of this article (10.1186/s13063-019-3644-5) contains supplementary material, which is available to authorized users.

## Background and rationale

Even though the prognosis after myocardial infarction (MI) has improved much over recent decades due to improved and faster revascularization, platelet inhibition, and device therapy [1, 2], a proportion of postacute MI (AMI) patients with additional risk factors remain at high risk, with 5-year mortality ranging from 15% to 45% depending on the population [3–5]. The major issue for future research in this population is to establish risk factors that identify patients remaining at high risk and in whom major adverse cardiac events (MACEs) can be prevented [6]. Clinical risk factors remain important and perform well but, as reviewed below, it is evident that discrimination can get better.

Data from the observational Cardiac arrhythmias and risk stratification after myocardial infarction (CARISMA) study showed that incident arrhythmias detected by an implantable cardiac monitor (ICM) in post-MI patients were more predictive for MACE than a large number of known demographical, clinical, and diagnostic risk parameters [7–10]. Due to the observational design of that study, it was not possible to deduct from the results whether diagnosing and treating the arrhythmias would improve the clinical course of the patients. Consequently, the Biomonitoring in patients with preserved left ventricular function after diagnosed myocardial infarction (BIO-GUARD-MI) study has been designed to answer this question.

### Risk stratification using conventional clinical risk factors: role of the CHADS-VASC score for risk stratification beyond LEVF

Left ventricular ejection fraction (LVEF) has come to play a dominant role in triaging patients towards high-risk or low-risk regimes. If LVEF is 35% or less, patients are implanted with an implantable cardioverter-defibrillator (ICD) due to the high risk of malignant arrhythmias. After ICD implantation, patients are not only followed with regular in-clinic controls, they are also continuously monitored by remote monitoring (RM) systems through the ICD. Therefore, beyond an improved prognosis by preventing sudden cardiac death, these patients may additionally benefit from RM through a decreased risk for hospitalization and death [11, 12].

In contrast, patients with relatively preserved left ventricular function are considered at low risk, and current treatment includes 1 year of dual antithrombotic therapy, after which only acetylic salicylic acid, beta blockers, and statins are continued, and the patients are followed in general practice. While their risk is certainly lower, this large difference in treatment and patient follow-up is not justified by the prognosis in these groups [13–15]. In a recent study including 1500 unselected consecutive patients with MI, only 20% of patients had an LVEF ≤ 40% [16]. The incidence of cardiovascular deaths or hospitalizations due to heart failure was 6% at 30 months, with 56% of these events occurring in patients with preserved LVEF. Hence, most events occur in the group of patients with relatively preserved ejection fraction but with other risk factors.

Multiple investigational noninvasive risk parameters have been developed and shown to stratify the risk in postinfarction patients beyond LVEF. These include signal-averaged electrocardiography (ECG) [17], T-wave alternans [18], and Holter parameters of baro-reflex sensitivity [19] and heart rate variability [20]. These parameters are not used clinically, mainly because no clinical benefit of any treatment has been shown for patients with these risk markers. Furthermore, they generally predict nonsudden (rather than sudden) death and the clinical response is not trivial [6]. In addition, the diagnostic risk markers derived from Holter monitoring, signal averaged ECG, T-wave alternans, or programmed stimulation study did not stratify the risk beyond clinical risk parameters [8].

The clinical prediction rules for estimating risk of stroke in patients with atrial fibrillation (the older CHADS_2_ score and the newer CHA_2_DS_2_-VASc score) were designed to estimate the risk of stroke in patients with atrial fibrillation. However, these scores contain the most important cardiovascular clinical risk factors and it is not surprising that there is a strong association between increasing scores and the risk of clinical arrhythmias as well as MACEs [21–25].

### Risk stratification and arrhythmia management using implantable devices

Recently, large randomized trials have shown that long-term monitoring of cardiac arrhythmias using RM in patients with cardiac devices may improve clinical outcome. The Implant-based multiparameter telemonitoring of patients with heart failure (IN-TIME) trial randomized 664 patients with ICDs or cardiac resynchronization therapy defibrillators (CRT-Ds) to RM or conventional in-clinic monitoring. There was a 30% reduction in the composite clinical (‘Packer’) score and a statistically significant reduction in all-cause mortality [11]. The majority of clinical alerts triggering reaction were arrhythmias, but the data collected did not allow for a precise correlation between physicians’ responses and outcome.

The Effectiveness and cost of ICD follow-up schedule with telecardiology (ECOST) trial of 433 ICD patients randomized to RM or no RM did not show an improvement in the rate of MACEs. However, a recent patient-level re-analysis found that RM reduced the endpoint of death or hospitalization for heart failure (combined hazard ratio at 12 months, 0.64; *P* = 0.007) in pooled data from the IN-TIME and ECOST trials [12], with both trials showing similar effect sizes.

In parallel, the Remote management of heart failure using implantable electronic devices (REM-HF) trial, a study of 1650 patients with ICD and CRT devices randomized to weekly RM transmissions or no RM, did not find an effect on the primary endpoint of all-cause death or cardiovascular hospitalization (hazard ratio, 1.01; *P* = 0.87) [26]. The differences in outcome between IN-TIME and ECOST on one side and REM-HF on the other can be plausibly attributed to differences in transmission rate (daily versus weekly) and other details in the study design [27].

In the observational CARISMA trial, 300 patients with an AMI and an LVEF < 40% were implanted with an ICM [7]. The study showed that arrhythmias documented on the ICM preceded 70% of MACEs, and cardiac arrhythmia was the most powerful predictor for a MACE. In fact, an unexpectedly high incidence of new-onset atrial fibrillation, second- or third-degree atrioventricular block, sinus bradycardia, ventricular tachycardia, and ventricular fibrillation were diagnosed by the ICM during the study. Almost 90% of these arrhythmias were asymptomatic, short lasting, and occurred at night, and hence would probably go undetected without the implanted ICM. Further investigation and treatment of these arrhythmias were, however, not part of the protocol, and whether intervention and treatment of ICM-documented arrhythmias could have changed the outcome remains unanswered.

The BIO-GUARD-MI study population selection criteria are based on these observations. The study enrolls patients with an LVEF > 35%. Little is known about the incidence of arrhythmia and the correlation to clinical outcome in this population, and the BIO-GUARD-MI study is therefore designed to investigate not only the incidence of arrhythmias according to different risk profiles, but also how arrhythmias are associated with major clinical events and whether management of these arrhythmias according to current guidelines influences the clinical course of the patients.

## Study objectives

The primary objective of the BIO-GUARD-MI study is to investigate whether the early diagnosis of cardiac arrhythmias provided by an ICM with automated daily RM and the consequent medical treatment will decrease the risk of MACEs in patients with previous AMI, LVEF > 35%, and a CHA_2_DS_2_-VASc risk score ≥ 4/5 (men/women). The study is powered to show a relative reduction of 25%.

Secondary objectives include the time to diagnosis of arrhythmias, each MACE component evaluated individually, the influence of each component of the CHA_2_DS_2_-VASc score on MACEs, and quality of life. Exploratory analyses will address the following interactions: 1) arrhythmias and clinical endpoints; 2) baseline conditions and clinical endpoints; 3) baseline conditions and arrhythmias; and 4) treatments and clinical endpoints.

## Study design

**Fig. 1:**
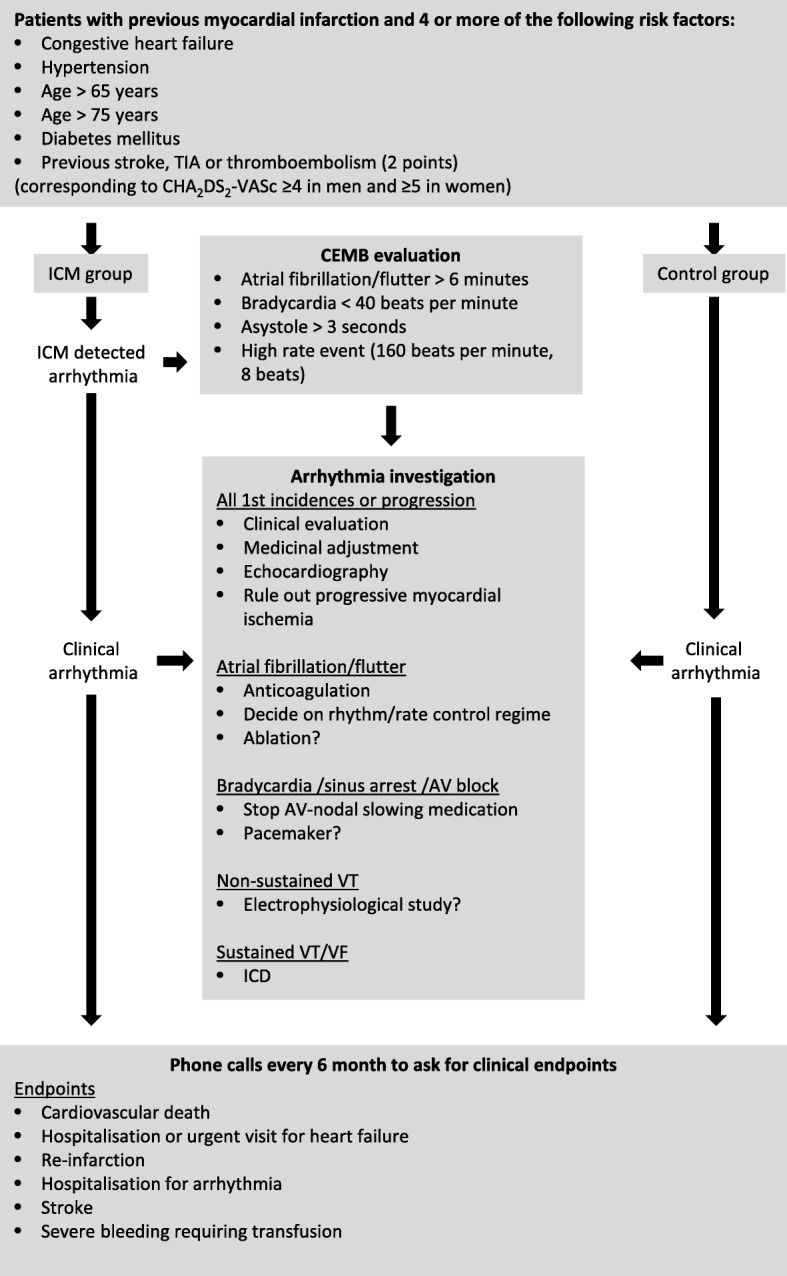
BIO-GUARD-MI flow diagram. AV atrioventricular, CEMB central electrocardiogram monitoring board, ICD implantable cardioverter-defibrillator, ICM implantable cardiac monitor, TIA transient ischemic attack, VF ventricular fibrillation, VT ventricular tachycardia

The BIO-GUARD-MI study is a multicenter, open, prospective, randomized controlled international study with an event-driven design. The investigational sites are hospitals with facilities for the treatment of AMI and experience in device treatment of arrhythmias. High-risk patients with recent or chronic MI are invited to participate. A flow diagram of the study is shown in Fig. 1. A spirit checklist can be found online (Additional file 1).

Patients who fulfill the enrollment criteria may be enrolled after conclusion of treatment for an AMI (if an ICD indication was excluded by a confirmed LVEF > 35%) or if they had a chronic MI. Investigators invite them to participate and collect written informed consent. Patients are randomized in a 1:1 ratio to receive implantation of an ICM or conventional treatment. The investigator receives the randomized allocation from a concealed computer-generated sequence stratified for the investigational site and ST-elevation MI (STEMI)/non-STEMI (NSTEMI) after enrollment. Both investigator and patient are aware of the randomization result. All patients are discharged to the typical post-MI follow-up. Neither ICM nor control group patients are scheduled to return to the implanting site for regular follow-up visits.

**Fig. 2:**
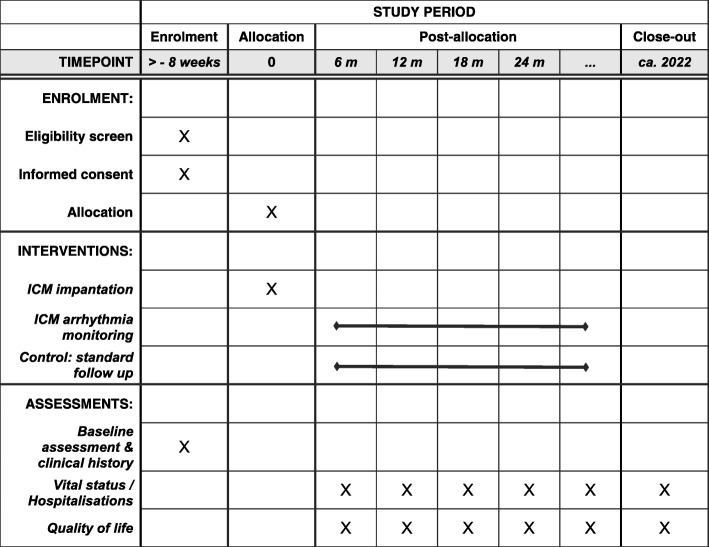
SPIRIT figure. ICM implantable cardiac monitor

To assess the primary endpoint, all patients will receive telephone calls every 6 months from an independent Clinical Research Organization. If they report that they have been hospitalized, the investigational site is notified and reports the adverse event based on documentation requested from the relevant hospital. The telephone calls will be conducted in a way so as not to interfere in the normal healthcare (i.e., the patient will receive no medical advice of any kind but will only be asked about events of the preceding period) (see also Fig. 2).

This study complies with the declaration of Helsinki and with ISO 14155. It is registered at clinicaltrials.gov (NCT02341534).

### Inclusion and exclusion criteria

Patient inclusion and exclusion criteria are presented in Table 1. Changes were made to the enrollment criteria in that a limitation to enrollment within 21 days of an AMI was removed to increase the eligible population and to also describe the incidence of arrhythmias very late after the MI. Furthermore, the CHADS_2_ score used in the initial protocol was later substituted with the CHA_2_DS_2_-VASc score which took over the role of CHADS_2_ in cardiovascular guidelines and clinical practice.

**Table 1:** Inclusion and exclusion criteria

Inclusion criteria	
History of MI according to guidelines:	
CHA_2_DS_2_-VASc score ≥ 4 in men/≥ 5 in women	
LVEF > 35% as estimated within 6 months before enrollment but after conclusion of acute MI treatment	
Patient accepts activation of home monitoring	
Patient has provided written informed consent	
Exclusion criteria	
Platelet count < 90,000 per mm^3^ or patients with hemorrhagic diathesis	
Permanent oral anticoagulation treatment for atrial fibrillation	
Indication for chronic renal dialysis	
Pacemaker or ICD implanted or indication for implantation	
Parkinson’s disease	
Life expectancy < 1 year	
Participation in another interventional clinical investigation during the course of the study, i.e. the participation in a noninterventional clinical investigation is allowed.	
Age < 18 years	
Woman who are pregnant or breast feeding	

*ICD* implantable cardioverter-defibrillator, *LVEF* left ventricular ejection fraction, *MI* myocardial infarction

### The implantable cardiac monitor

The BioMonitor 2 (Biotronik SE & Co. KG, Berlin, Germany) or successor devices will be used [28]. It is a subcutaneous ICM that continuously monitors the heart rhythm. It automatically records a subcutaneous ECG (sECG) when triggered according to certain criteria, as shown in Table 2. The expected service time is 4 years; if a device battery expires during the study, it will be replaced. The device automatically transmits once per day up to six uncompressed full-length sECG snapshots to the Home Monitoring Service Center via Biotronik Home Monitoring^®^ [29]. The physician can assess the message content on a secure website.

**Table 2:** Recommended responses to detected arrhythmias, the rationale, and literature behind the rationale

Arrhythmia	Definition according to ICM programming	Recommended investigations and treatments	Rationale
First incidence or sudden progression in burden of any arrhythmia		ECG to evaluate QRS width and other intervals, ischemia, etc. Echocardiography to exclude progressive HF or valve disease. Consider coronary angiogram if symptoms exceed or cannot be explained by arrhythmia severity	BIO-GUARD-MI is based on data from the CARISMA trial [7]. In that study, any arrhythmia including sinus bradycardia, AV block, AF, nonsustained VT, and sustained VT/VF was associated with an increased risk of any endpoint including re-infarction, stroke, progressive HF and death. Hence, we recommend that any patient be evaluated for progressive HF or recurrence of symptomatic ischemic heart disease
AF or atrial flutter < 5.5 h	RR variability > 12.5% or detected as HVR (> 160 bpm for 8 cycles) for > 6 min, but lasting < 5.5 h total/24 h (includes bigeminy rejection)	Beta blocker	In a large registry-based analysis, beta blockers were associated with a better prognosis in patients with AF [30], after cardiac surgery [31] and renal disease [32]. In comparison, calcium antagonists may be equally efficient for symptom relief and rate control [33], but there is currently a paucity of knowledge for best medicinal rate control methods [34]. Based on this rationale, beta blockade is the recommended primary medication for MACE prevention and for rate control in new-onset paroxysmal AF in this population
		Initiation of anticoagulation therapy according to patient profile and wishes	Current consensus on device-detected AF states that anticoagulation can be considered but does not necessarily need to be initiated in high-risk patients if daily episodes last < 5.5 h/day [35]. All patients in the BIO-GUARD-MI study have a CHA_2_DS_2_-VASc score indicating a high stroke risk
AF or atrial flutter > 5.5 h	RR variability > 12.5% or detected as HVR (> 160 bpm for 8 cycles) > 5.5 h total/24 h (includes bigeminy rejection)	Initiate anticoagulation therapy	Current guidelines recommend initiation of anticoagulation treatment if the total duration of daily episodes of AF exceeds 5.5 h/day [35].
		DC cardioversion if appropriate. Plan for rhythm and/or frequency management strategy. Rhythm management strategy is encouraged unless there are contraindications to this, or the patient is unwilling. Radiofrequency ablation is preferred over drug treatment as long-term rhythm management	Even though rate control is not inferior to rhythm control, there is an advantage if sinus rhythm can easily be restored [8]. In a large Cochrane-based review, there seems to be a slightly higher mortality when choosing a rhythm control [36], but this seems to be due to the use of antiarrhythmic drugs, whereas catheter ablation is consistently associated with an improved outcome in high-risk patients [37, 38]
		Optimize antihypertensive treatment	Antihypertensive treatment has been repeatedly shown to lower the incidence of AF [39], and controlling systolic blood pressure over time seems to be key [40].
Bradycardia	< 40 bpm for ≥ 10 s	If < 40 bpm and symptomatic, or < 30 bpm regardless of symptoms, patient should be evaluated for optimization of medical treatment and pacemaker therapy	Even though based on sparse literature, current guidelines do not recommend pacemaker implantation in asymptomatic sinus bradycardia [41]. In the CARISMA trial, sinus bradycardia was associated with an adverse outcome, mainly due to an association with progressive HF [7]. Hence, we recommend thorough evaluation of symptoms, heart function, and coronary circulation in progressive sinus bradycardia, particularly with heart rate < 30 bpm. There are currently no randomized or larger observational trials to support management of these events
Sinus arrest	Pause lasting > 3 s	For asymptomatic pauses > 6 s or symptomatic pauses > 3 s, adjust medicine accordingly and evaluate for pacemaker therapy	Even though such pauses are considered benign and not associated with an adverse outcome, pacemaker implantation can be considered for prevention of syncope [41]. Sinus arrest was not associated with an adverse outcome in CARISMA [7]
AV block type 2 or 3	< 40 bpm for ≥ 10 s or pause > 3 s	Pacemaker implantation for high-degree AV block persisting after AV nodal slowing medications.	For patients with AV block, there is class I indication for pacemaker implantation regardless of symptom/arrhythmia correlation [41]
		If LVEF ≤ 40% a CRT-P is recommended in patients with an expected right ventricular pacing burden > 50%	Studies have shown that biventricular pacing in patients with symptomatic HF and high ventricular pacing burden can prevent HF events compared with right ventricular pacing. The precise indications in terms of ejection fraction and pacing burden are not specified in current guidelines. For the purpose of the present study, we consider the specified cut-offs reasonable
Nonsustained VT	HVR > 160 bpm for 8 cycles	If LVEF ≤ 35%, EPS (MADIT-I) is recommended	An EPS has been shown to identify high-risk patients with previous myocardial infarction that will benefit from an ICD because of high risk of malignant arrhythmias [42, 43]
		If symptomatic after beta blocker treatment, radiofrequency ablation is recommended	Multiple studies have shown that radiofrequency ablation effectively treats ventricular ectopy and tachycardia [44]. While an effect on mortality has never been proved, ablation in patients with ventricular burden > 20% may restore left ventricular function in the setting of HF [45]
Sustained VT or VF	HVR > 160 bpm for 8 cycles	Implantation of ICD or CRT-D. Radiofrequency ablation is preferred over drug treatment as long-term rhythm management	Sustained VT > 30 s or VF gives indication for an ICD according to guidelines [42, 46]. If ECG has QRS > 150 ms or observations on the ICM indicates risk of high right ventricular pacing percentage (> 40%), a CRT-D device should be implanted [41]

*AF* atrial fibrillation, *AV* atrioventricular, *CRT-D* cardiac resynchronization therapy defibrillator, *CRT-P* cardiac resynchronization therapy pacemaker, *DC* direct current, *ECG* electrocardiogram, *EPS* electrophysiological study, *HF* heart failure, *HVR* high ventricular rate, *ICD* implantable cardioverter-defibrillator, *ICM* implantable cardiac monitor, *LVEF* left ventricular ejection fraction, *MACE* major adverse cardiac event, *RR* cycle length, *VF* ventricular fibrillation, *VT* ventricular tachycardia

### Arrhythmia diagnosis, documentation, and response

The BIO-GUARD-MI study has been designed to track and document the time-wise response of the investigators to arrhythmia events. A large effort has been made to ensure that current guidelines are followed where appropriate. In some instances the guidelines do not provide precise recommendations and, in these cases, recommendations have been based on previous literature and observations from the CARISMA study. Recommended responses to detected arrhythmias and the rationale and literature behind the responses are provided in Table 2.

### Central electrocardiogram monitoring board

The central electrocardiogram monitoring board (CEMB) receives alerting e-mails from the Home Monitoring Service Center and reviews all sECGs. It discards detections of noise or artifacts, and if any events fulfill predefined criteria the responsible physician is notified by e-mail. At the same time, the CEMB enters the event in the study database to ensure correct recording of the event, including the precise time of the sECG transmission to allow the evaluation of the time to the investigator’s reaction. The CEMB also provides a centralized follow-up on the investigator’s assessment, who is responsible for checking and adjudicating on the arrhythmias immediately but at the latest within 7 days after receiving an arrhythmia notice from the CEMB. However, the CEMB does not support ECG interpretation or medical decisions for study participants.

### Endpoints

The primary endpoint is the time from randomization to the first MACE during the clinical investigation. MACEs comprise the following events: 1) cardiovascular death; 2) worsening of the status of the patient due to heart failure, requiring acute unscheduled hospitalization or urgent visit; or 3) unscheduled cardiovascular hospitalization due to arrhythmia, acute coronary syndrome, stroke, major bleeding, or systemic embolism.

This broad definition of the primary endpoint was chosen due to the basic assumption that all the items are increased in post-MI patients and that acute arrhythmias can be predictive for all of them. Therefore, treatment of the patients after acute arrhythmias may improve any of the items. Hospitalization that result from events detected in the ICM are by definition scheduled and are not counted as endpoints. Major bleeding was included to capture possible side effects of anticoagulation treatment after detection of AF.

Subgroups are predefined according to the following characteristics at enrollment for exploratory analyses with respect to the occurrence of the predefined arrhythmias, the primary endpoint, and other predefined outcomes: 1) all individual CHA_2_DS_2_-VASc-score components; 2) men versus women; 3) CHA_2_DS_2_-VASc-score (≤ 4 in men/≤5 in women versus ≥5 in men/≥6 in women); 4) age (< versus ≥ median); 5) LVEF (< versus ≥ median); 6) body mass index (underweight or normal or overweight versus obese, cut-off 30 kg/m^2^); 7) history of atrial fibrillation (yes versus no); 8) NSTEMI versus STEMI; 9) ‘early’ (within 40 days of most recent AMI) versus ‘late’ enrollment; and 10) history or presence of kidney failure (yes versus no).

### Boards and committees

A Data Safety Monitoring Board (DSMB) will regularly review accumulating study data to address patient safety and ethical issues of the study. The DSMB will perform the two interim analyses and the final analysis of the primary endpoint. Based on the interim data, the DSMB will give the request to stop the clinical investigation for superiority or give a recommendation to the steering committee and the sponsor whether to continue the clinical investigation as planned, to adapt the sample size, or to stop the clinical investigation for safety reasons or for futility.

The blinded Endpoint and Adverse Event Committee will analyze adverse events with respect to the specified endpoint criteria and will adjudicate if a primary or secondary endpoint has been met.

Members of both bodies cannot participate in the study as investigators.

### Data collection

Data will be entered into a password-protected internet-based Clinical Data Management System purposely designed by the sponsor and hosted by MedNet Solutions (MN, USA). Data will be monitored by trained employees of the sponsor or subcontracted CROs.

### Statistical design and analysis

The primary hypothesis will be tested with the Kaplan–Meier method by a log-rank test stratified for STEMI/NSTEMI after enrollment. All analyses will be conducted according to the intention-to-treat principle. Patients who exit the study prematurely will be included in the analysis until their exit. Details will be defined in a statistical analysis plan.

The study is designed to allow for early discontinuation in case of superiority or futility and to adapt the sample size in case of positive results but insufficient power to meet the statistical significance with the initially planned sample size. The study is designed as a three-stage adaptive group sequential test procedure according to O’Brian Fleming with survival endpoint.

Survival-analysis techniques (Kaplan–Meier, log-rank test, Cox regression, hazard ratio and confidence interval) will be used to compare the incidence of all survival data endpoints between the groups. Interaction with subgroup variables and other covariates will also be analyzed by a Cox proportional-hazards model. For two-sided and one-sided statistical tests, *P* values < 0.05 and < 0.025 will be considered statistically significant, respectively. No adjustments for multiplicity will be undertaken, and all findings based on prespecified hypotheses as well as post-hoc analyses such as multivariate analyses will express only supportive evidence for the primary hypothesis. Statistical testing will be conducted with SAS software, version 9.4 or later (SAS Institute).

### Sample size

It is expected that the risk of a first MACE at 1 year is reduced by 25% in the ICM group compared with the control group. A one-sided log-rank test is performed in the context of a confirmatory analysis of the primary hypothesis at every interim analysis and the final analysis. A total of 372 patients with events are needed to maintain the overall 2.5% significance level. The interim analyses and the final analysis will be conducted with 124, 248, and 372 patients with at least one MACE during the clinical investigation, respectively. The statistical power of the final analysis is 80%. A drop-out of 5% of patients per year is expected.

The total number of patients who will be included will depend on the observed endpoint rate and on enrollment speed, since the first enrolled patients are followed for a longer period if enrollment is slower. Current estimates indicate that up to 1400 patients will be enrolled.

### Risk of bias

Blinding would have been possible if control patients received an inactivated ICM. The decision against this option was based on both the invasiveness of the ICM implantation and the possibility that general practitioners (GPs) or local cardiologists might treat patients with an ICM (as they do not know it is inactive) differently than they would standard post-MI patients.

Thus, the risk of bias was reduced by other measures. All unnecessary contacts between patients in the ICM group and the investigational site are avoided by the study design, so that a preferential treatment of patients by investigators is unlikely. Patients are followed by GPs or local cardiologists (as per regional routine) and do not return to the investigational site except for the treatment of arrhythmias. GPs are asked to send the study patients of both groups to the investigational site for treatment of arrhythmias that are recorded conventionally if tertiary level care is required. Furthermore, because the RM system is known to be very reliable, patients are only contacted by the investigational sites for rare long-lasting transmission gaps (> 40 days) [29].

## Discussion

There is little doubt that using implantable monitors to continuously monitor biological functions will increase in the future and to some extent reduce the need for in-clinic controls. However, several open questions remain, including what to monitor and how frequent and how sensitive the monitoring should be.

While tailored patient management guided by RM has been tested in many groups of patients, the focus has been driven by the availability of technology and has concentrated on patients with diabetes, chronic obstructive pulmonary disease (COPD), chronic heart failure, and hypertension. A search on ClinicalTrials.gov shows that most trials investigating telemedicine to a large extent still involve these patient groups. Hence, at least in the near future, heart and pulmonary function, blood pressure, and simple metabolic measures seem to dominate telemedicine.

RM results in better treatment control in patients with diabetes [47] and hypertension [48], whereas this effect has been more difficult to prove in patients with COPD [49]. A rationalistic interpretation could be that monitoring is more promising in conditions that present with little or no symptoms, such as hyperglycemia and high blood pressure, whereas COPD exacerbations present quite acutely. Another key element for successful RM seems to be a simple and easily implementable response to the information obtained by RM. The recent CardioMEMS heart sensor allows monitoring of pressure to improve outcomes in NYHA class III heart failure patients (CHAMPION) trial involved continuous measurement of the pulmonary wedge pressure. The response was a simple, predefined titration scheme for guideline-recommended heart failure medication dosage which resulted in a significant decrease in hospitalizations due to heart failure [46, 50]. In contrast, some larger trials involving monitoring of multiple biological measures have shown neutral results [51, 52], while modifications in design can make a difference [53].

ICMs have decreased in size and improved in their signal quality. Their sensitivity for detecting brady arrhythmias and ventricular high-rate episodes is very good, but specificity is limited due to undersensing and noise. For this reason, the CEMB was instituted to filter these episodes. The sensitivity for shorter regular episodes of atrial fibrillation is lower, but it is unlikely that the device will miss longer lasting (e.g., hours to days) episodes of atrial fibrillation. Hence, the sensitivity to identify patients with arrhythmias is very good [28]. Due to their subcutaneous positioning, they cause a very low risk, and they do not limit the patient’s activities. Remote monitoring of cardiac implants, especially ICDs, has been established for a long time, is well accepted by the majority of patients, and is state of the art in ICMs these days.

Cardiac arrhythmias frequently start as asymptomatic, shorter lasting, and nightly events [7], and hence seem to be well suited for RM. The downside is that, since cardiac arrhythmias are linked to an increased risk of multiple outcomes, a response is not always simple and straightforward. The BIO-GUARD-MI trial represents the first attempt to simplify the response to the rather complex nature of cardiac arrhythmias and, if successful, we believe the trial can significantly advance the field of clinical telemedicine and contribute to improved patient care and prevention of hospitalization.

## Trial status

The trial is currently recruiting. The patient recruitment phase started in August 2015 and is expected to end in August 2021. The current protocol version is 4.0, 28 April 2017. All amendments were submitted to the ethics committees and, if changes were significant, were approved by them. All investigators were trained on the amendments.

## Additional file

Additional file 1: SPIRIT 2013 checklist: recommended items to address in a clinical trial protocol and related documents. (PDF 42 kb)

## Data Availability

The data that support the findings of this study will be available from Biotronik, but restrictions apply to the availability of these data, and so are not publicly available. Data may, however, be available upon reasonable request and with permission of the steering committee and Biotronik.

## Abbreviations

AMI: Acute myocardial infarction
CEMB: Central electrocardiogram monitoring board
CHA_2_DS_2_-VASc: Clinical prediction rules (newer) for estimating the risk of stroke in patients with atrial fibrillation
CHADS_2_: Clinical prediction rules (older) for estimating the risk of stroke in patients with atrial fibrillation
COPD: Chronic obstructive pulmonary disease
CRT-D: Cardiac resynchronization therapy defibrillator
DSMB: Data Safety Monitoring Board
ECG: Electrocardiography
ICD: Implantable cardioverter-defibrillator
ICM: Implantable cardiac monitor
LVEF: Left ventricular ejection fraction
MACE: Major adverse cardiac event
MI: Myocardial infarction
RM: Remote monitoring
sECG: Subcutaneous electrocardiography
STEMI: ST-elevation myocardial infarction
